# Fine Mapping of Rice Specific *MR1*, a Gene Determines Palea Identity

**DOI:** 10.3389/fpls.2022.864099

**Published:** 2022-05-24

**Authors:** Wei Xie, Wei Liu, Xiaoqi Yu, Dali Zeng, Deyong Ren

**Affiliations:** State Key Laboratory of Rice Biology, China National Rice Research Institute, Hangzhou, China

**Keywords:** rice (*Oryza sativa* L.), *mr1* mutant, palea, organ origin, grain size and quality

## Abstract

The hull (palea and lemma) is the specific organ of grass florets. Although many genes related to the hull development have been cloned, the genetic mechanisms behind the development are still unclear, and the evolutionary relationship has different explanations and heated arguments between the palea and lemma. In this study, we found a specific *mr1* mutant with a reduced palea, showing an enlarged mrp and degraded bop. Phenotype observations and molecular evidences showed that the bop was converted to the mrp-like organ. Our findings first reveal that the bop and mrp are homologous structures, and the palea and lemma are the same whorl floral organs. *MR1* may prevent the transformation of the bop into mrp by regulating the expressions of hull identity genes. Meantime, the *mr1* mutant showed altered grain size and grain quality, with defective physical and chemical contents. *MR1* was controlled by a single recessive gene and was finally located on chromosome 1, with a physical distance of 70 kb. More work will be needed for confirming the target gene of *MR1*, which would contribute to our understanding of grain formation and the origin between the lemma, bop, and mrp.

## Introduction

The rice hull, such as palea and lemma, is the unique floral organ of the outer whorls in grass (Xu et al., 2021). They are located in the outer layer of floret structure, which can provide photosynthetic products for internal organs in the early stage, and protect seeds from diseases and insect pests after ripening. The hull size is also one of the determinants of seed size. Therefore, the hull development is very important for the formation of rice yield and quality.

The flowers of typical eudicots are composed of sepals, petals, stamens, and pistils. According to the genetic and molecular analyses of *Arabidopsis thaliana* and *Antirrhinum majus*, Coen and Meyerowitz proposed an ABC model of floral organ characteristics in the early 1990s (Coen and Meyerowitz, 1991; Weigel and Meyerowitz, 1994). The classical ABC model suggests that there are three kinds of genes A, B, and C to control the development of four whorls floral organs, the same class of genes control the two adjacent whorls. That is to say, the sepals, petals, stamens, and pistils of four floral organs are determined by A, AB, BC, and C genes, respectively (Yanofsky et al., 1990; Mandel et al., 1992; Goto and Meyerowitz, 1994). Gradually, due to the identification of class D genes that regulate ovule development and the class E genes that regulate the development of internal three whorls of floral organs, ABC model is further extended to ABCDE model (Colombo et al., 1995; Pelaz et al., 2000; Theissen and Saedler, 2001). With our deep-going understanding of the flower development of monocots, we found that in monocots such as rice, the shape and function of the stamens and pistils of the inner whorls are similar to those of eudicots, and the ABCDE model is also applicable to them; however, the shape and function of the hull and lodicules of the outer whorls are quite different from those of eudicots.

In *Arabidopsis*, the development of the outer whorls of floral organs is mainly regulated by class A and E genes (Coen and Meyerowitz, 1991; Angenent et al., 1995; Pelaz et al., 2000). Class A genes include *APETALA1* (*AP1*) and *APETALA2* (*AP2*), they specifically regulate the development of sepals and petals in the outer two whorls of floral organs. Mutations in the class A genes will lead to the homeotic transformation from sepals and petals to pistils and stamens (Angenent et al., 1995). There are four class E genes in *Arabidopsis*, *SEPALLATA1*/*2*/*3*/*4* (*SEP1*/*2*/*3*/*4*). At first, it is found that *SEP1*/*2*/*3* redundantly regulates the characteristics of three inner whorls, and later it is found that *SEP1*/*2*/*3* also redundantly regulates the development of sepals together with *SEP4* (Pelaz et al., 2000).

In rice, there are class A and E genes homologous to *Arabidopsis*. *OsMADS14*/*15*/*18*/*20* are the orthologous genes of *AP1* in *Arabidopsis*. *OsMADS14* is mainly expressed in the inflorescence and developing seeds (Pelucchi et al., 2002), the overexpression of *OsMADS14* can lead to a significant advance in rice flowering. The *degenerative palea* (*dep*) mutant is caused by a single nucleotide mutation of *OsMADS15* gene, which shows that the lemma is elongated and the body of palea (bop) is degenerated, leaving only two marginal regions of palea (mrp), which indicates that *OsMADS15* is necessary to maintain the bop and the development of the lemma (Wang et al., 2010). The overexpression of *OsMADS18* leads to the early flowering and the early initiation of axillary meristem, which indicates that *OsMADS18* can initiate differentiation of branches and leaves (Fornara et al., 2004). *OsMADS20* is only expressed in the stems and developing seeds, and it is significantly different from the expression pattern of class A genes in *Arabidopsis*. Class E genes include two main types: *SEP1*/*2*/*4* (*LOFSEP*) and *SEP3* (Malcomber and Kellogg, 2005). *SEP1*/*2*/*4* genes include *OsMADS1*/*LHS1*, *OsMADS5*, and *OsMADS34*, and *SEP3* genes include *OsMADS7*/*OsMADS45* and *OsMADS8*/*OsMADS24*. *OsMADS1* plays a key role in the late developmental stages of floral organs, and it first expressed in the meristem of spikelet (Chung et al., 1994; Prasad et al., 2001). Among several mutants of *OsMADS1* gene, the palea and lemma elongated in varying degrees, indicating that *OsMADS1* plays an important role in the development of palea and lemma (Jeon et al., 2000). The complete loss of *OsMADS1* function resulted in the complete homeotic transformation of the three inner organs (lodicules, stamens, and pistils) into the palea and lemma-like structures (Agrawal et al., 2005). The sterile lemma of *osmads34* mutant was elongated, while the palea and lemma were normal, but in the *osmads34* and *osmads1* double mutant, the palea and lemma were more elongated and leafed than the *osmads1* single mutant, which indicated that *OsMADS34* and *OsMADS1* redundantly regulate the development of palea and lemma (Gao et al., 2010). The overexpression of *OsMADS5* promoted the early flowering phenotype, but did not affect the morphology of flower (Jeon et al., 2000). OsMADS7 and OsMADS8 can interact with other proteins such as OsMADS13, OsMADS16, and OsMADS18 (Favaro et al., 2003; Kater et al., 2006). And *OsMADS7* and *OsMADS8* have similar interaction patterns with *OsMADS1*. But it is still not clear whether the genes *OsMADS5*/*7*/*8* play a role in the regulation of palea and lemma development.

In this study, we identified a mutant with abnormal palea development, named *marginal region 1* (*mr1*). The *mr1* mutant showed a defective fusion palea with an enlarged mrp and a degraded bop, first revealing that the bop and mrp are homologous organs. The *mr1* mutant also showed altered grain size and quality, with defectively physical and chemical contents. Further, we performed genetic analysis and fine mapping of the *mr1* mutant traits, which tried to detect the *MR1* gene and its function. The present and future work will contribute to our understanding of grain formation and organ origin.

## Materials and Methods

### Plant Materials

The *mr1* mutant was identified in the ethyl methanesulfonate (EMS) mutant population of Shuangkezao (SKZ) (cv. *indica*), and the mutant character was inherited stably after multiple generations of self-bred and selection. The *mr1* mutant was crossed with Nipponbare to construct a mapping population. The resulting F_1_ grains were sown and self-crossed to construct the F_2_ population, which used for gene mapping and genetic analysis. All these materials were planted under natural conditions in the paddy fields of China National Rice Research Institute.

### Morphological and Histological Analysis

In the flowering stage, we selected the fresh spikelets of wild-type and *mr1* mutant and dissected them with tweezers, then analyzed the phenotypic characteristics of them by NIKON SMZ1500 stereoscope.

Paraffin sections were used for histological analysis, according to the method of Ren et al., 2019b. We selected the spikelets of wild-type and *mr1* mutant at the heading stage and fixed them in mixed solution (50% absolute ethanol, 0.9 mol/L acetic acid, and 3.7% formaldehyde) at 4°C for more than 16 h, and dehydrated them with different concentrations of ethanol. These materials were then infiltrated with xylene and embedded in paraffin. A rotary microtome was used to cut the paraffin into 8 μm thick pieces and the slides were put at 42°C for several days. We chose some perfect slides to stain with safranine and fast green, and then dehydrated them with different concentrations of ethanol. These stained slides were infiltrated with xylene and covered with neutral resins. At last, we observed these slides under an optical microscope.

### Scanning Electron Microscopy

According to the method described by Mizukami and Ma, 1992, the scanning electron microscopy (SEM) analysis was performed. We selected the young spikelets of wild-type and *mr1* at the stage of floral organ differentiation and fixed them in 3% glutaraldehyde solution at 4°C for about 24 h. Then the materials were dehydrated with different concentrations of ethanol, and incubated in the mixture of ethanol and isoamyl acetate for 1 h. The materials were dried and wrapped with gold powder. These coated samples were finally observed under a HITACHI S-3500 SEM with a −30°C cooled stage.

### Fine Mapping of the Mutant Gene

Among the progenies from the cross between the *mr1* mutant and Nipponbare, we selected a total of 558 F_2_ plants which were showed the mutant phenotype for mapping of the *MR1* gene (Supplementary Table 1). The simple sequence repeat (SSR) markers we used were obtained from the GRAMENE website.^1^ And for fine mapping, we developed more new in/del primers by comparing the sequences of the *indica* 93-11 and *japonica* Nipponbare. Primer sequences used are listed in Supplementary Table 2.

### RNA Extraction and Gene Expression Analysis

The roots, culms, leaves, internodes, inflorescences at various developmental stages, and all floral organs (pistils, stamens, lodicules, paleae, and lemmas) of the wild-type and *mr1* mutant were collected, the total RNA was extracted from these tissues or organs using the RNeasy Plant Mini Kit (Axygen). The 2 μg total RNA was reverse transcribed to cDNA by using the Reverse Transcriptase Kit (Invitrogen) with genomic DNA eraser (Takara) in a 20 μl reaction volume. For the quantitative reverse transcription-PCR (qRT-PCR) analysis, the cDNA samples were used as templates after being diluted 10-fold. The qRT-PCR was carried out on a StepOne-Plus System (Applied Biosystems) using the SYBR Green PCR Mix Kit (Applied Biosystems) (Ren et al., 2019b). At least three biological replicates were carried out, and the relative expression levels were quantified using a relative quantitation method. Primer sequences used for qRT-PCR are listed in Supplementary Table 2.

### *In situ* Hybridization

Healthy young panicles from the wild-type and *mr1* mutant were put in 70% mixed solution (50% absolute ethanol, 0.9 mol/L acetic acid, and 3.7% formaldehyde), then dehydrated in gradient concentrations of ethanol and xylene, and finally embedded in paraffin. Hybridization and immunological detection of these samples were performed as previous description (Ren et al., 2018). The probes were prepared and labeled by a DIG RNA Labeling Kit from Roche according to the manufacturer’s recommendations. Primer sequences used for *in situ* hybridization are listed in Supplementary Table 2.

### Analysis of Grain Quality

The mature grains were air-dried at room temperature and then were crushed to rice powder for physicochemical properties analysis. The total starch content was examined by using a starch assay kit (Megazyme) (Cai et al., 2018). The amylose content measurement was performed according to the description by Liu et al. (2009), and the total protein content measurement was performed according to the description by Kang et al. (2005). To analyze the gelatinization temperature of the samples, we used a differential scanning calorimeter (DSC1). First, we put 5 mg rice powder into an aluminum cup and fixed it with 10 μl distilled water. Then we used a machine to seal the cup. At last, we put the cup into the DSC1, and it run under the 10°C min^–1^ heating rate and in the temperature range of 35–100°C. According to the method described by Cai et al. (2018), we examined the swelling and gelatinization characteristics of endosperm starch in urea solutions. All measurements contained three biological replications. Analysis of variance (ANOVA) was used to test whether there are significant differences between these samples.

## Results

### Phenotype of the *mr1* Mutant

**FIGURE 1:**
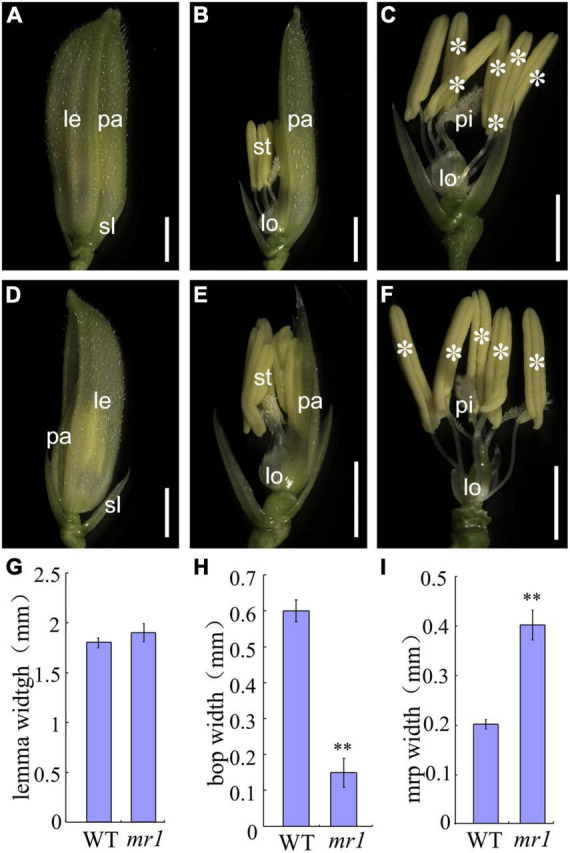
Phenotypes of wild-type and *mr1* florets. **(A)** Wild-type floret. **(B)** Wild-type floret without lemma. **(C)** Wild-type floret without lemma and palea. **(D)** *mr1* floret. **(E)** *mr1* floret without lemma. **(F)** *mr1* floret without lemma and palea. **(G)** Lemma width. **(H)** Bop width. **(I)** mrp width. le, lemma; pa, palea; sl, sterile lemma; lo, lodicule; st, stamen; pi, pistil. Asterisk indicates the stamen. Bars, 2 mm in **(A–F)**. **Significant difference at *p* < 0.01 compared with the wild-type by Student’s *t*-test. Error bars indicate SD.

In the wild-type, the floret includes a lemma, a palea, two lodicules, six stamens, and a pistil from outer to inner (Figures 1A–C). At the seedling and vegetative stage, the phenotype of the *mr1* mutant was normal, and the flowering period was also not different from that of wild-type. However, the *mr1* mutant appeared abnormal after heading stage. Compared to the wild-type, the *mr1* mutant showed reduced paleae, and it was prone to splitting (Figures 1A,B,D,E), but the lemma was not altered (Figures 1A,B,D,E). Meantime, the stamens of *mr1* mutant were reduced and no differences were found between the wild-type and *mr1* mutant in the other floral organs (Figures 1C,F). According to our measurements and statistics, the palea, bop and mrp of *mr1* mutant were narrower than that of wild-type, and the lemma had no significant changes (Figures 1G–I).

**FIGURE 2:**
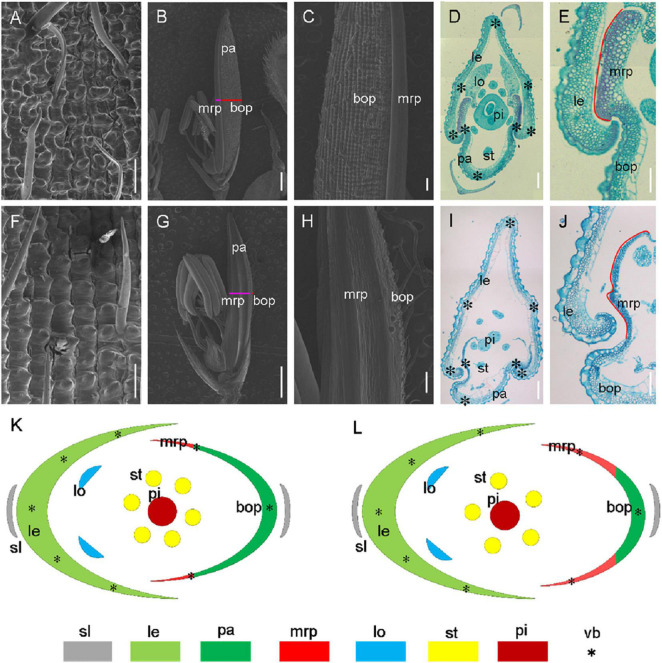
Histological analysis of hull in the wild-type and the *mr1* mutant. **(A)** Epiderm of the wild-type lemma. **(B)** Epiderm of the wild-type palea. **(C)** Enlarged palea in the wild-type. **(D)** Transverse section of wild-type floret. **(E)** Interlocked structure of wild-type hull. **(F)** Epiderm of the *mr1* lemma. **(G)** Epiderm of the *mr1* palea. **(H)** Enlarged palea in the *mr1* mutant. **(I)** Transverse section of *mr1* floret. **(J)** Interlocked structure of *mr1* hull. **(K)** Diagram of wild-type spikelet. **(L)** Diagram of *mr1* spikelet. le, lemma; pa, palea; lo, lodicule; st, stamen; pi, pistil; bop, body of palea; mrp, marginal region of palea; sl, sterile lemma; vb, vascular bundle. Black asterisks indicate the vascular bundles. Red lines indicate the mrp. Bars, 100 μm in **(A,E,F,J)**; 1 mm in **(B,G)**; 200 μm in **(C,D,H,I)**.

For histological analysis, we further carried out scanning electron microscopy (SEM) and paraffin sections for the florets of wild-type and *mr1* mutant (Figure 2). We found that in the normal floret, there were five vascular bundles in the lemma, and there were three in the palea (Figures 2A–E,K). And the palea comprised of two mrps and a body of palea (bop), which was different from the lemma (Figures 2B–E,K). As the outer whorls of floral organs, palea and lemma had their distinctive cellular morphology (Figures 2A–E). They both comprised of silicified cells (sc), non-silicified cells (nsc), fibrous sclerenchyma (fs), and spongy parenchymatous cells (spc) (Figures 2D,E,K). In addition, the mrp differed from the bop, showing a distinctive smooth epidermis but lacking silicified thickening epicuticular cells (Figures 2B–E,K). Because of this unique mrp, the lemma and palea appeared a well-interlocked structure (Figures 2B–E,K).

In the *mr1* mutant, the mrp was enlarged, and the bop was reduced compared with the wild-type (Figures 2F–J,L). However, the number of vascular bundles was not altered in the *mr1* palea, and the *mr1* lemma appeared normal vascular bundles (Figure 2I). And the reduced palea resulted in the poor interlocked between the mrp and lemma, so the hull was prone to splitting (Figures 2I,J,L). These results indicated that in the *mr1* mutant, the lemma kept normal phenotype, while the palea was significantly reduced due to the decrease of bop and increase of mrp. In addition, no obvious histocytological defects were observed for three inner whorls of floral organs except stamen number in the wild-type and *mr1* mutant (Figures 2B–D,I,J,L).

### Analysis of Floral Organ Development at the Early Stage

**FIGURE 3:**
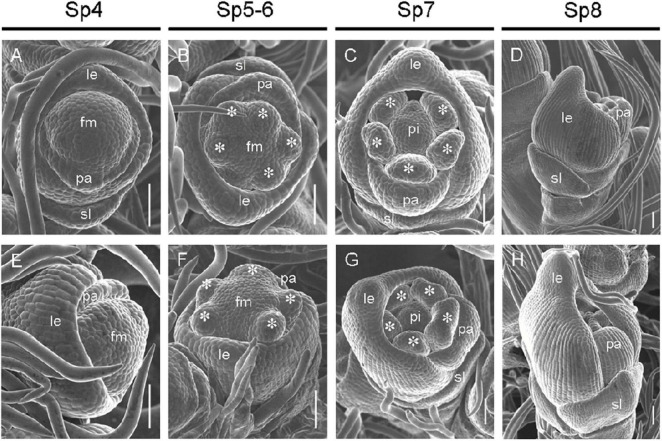
Florets at early developmental stages in the wild-type and the *mr1* mutant. **(A–D)** Wild-type floret. **(A)** Sp4. **(B)** Sp5-6. **(C)** Sp7. **(D)** Sp8. **(E–H)** *mr1* floret. **(E)** Sp4. **(F)** Sp5-6. **(G)** Sp7. **(H)** Sp8. fm, floral meristem; sl, sterile lemma; le, lemma; pa, palea; pi, pistil. Asterisk indicates the stamen. Bars, 50 μm.

The development of early differentiation stages of wild-type and *mr1* mutant florets was observed by SEM, the division of developmental stages we used refers to Ikeda et al. (2004). At the Sp4 stage, the primordia of lemma and palea of the wild-type floret began to develop, the primordia of lemma developed earlier, with a bulge on the top; and the lemma and palea were semicircular, and the marginal tissues were interconnected to form a circular structure, which held the floral meristem inside (Figure 3A). At this stage, no obvious differences were found between the wild-type and *mr1* mutant (Figure 3E). During the Sp5 to Sp6 stages, the stamen primordia of the wild-type florets began to differentiate, and the visible spherical stamen primordia were formed synchronously except the stamen adjacent to the lemma (Figure 3B). The primordia of lemma and palea further differentiated, and their marginal tissues began to interlock (Figure 3B). We also observed that the *mr1* palea was smaller than the wild-type in these stages (Figure 3F). At the Sp7 stage, the primordia of lemma and palea of the wild-type developed normally and presented a semi-closed state, wrapping the primordia of inner floral organs (Figure 3C). At Sp8, the primordia of lemma and palea of the wild-type was completely closed, and the inner whorls organs were completely wrapped (Figure 3D). Whereas, during the Sp7 and Sp8 stages, the *mr1* florets showed significantly smaller palea than that of the wild-type (Figures 3G,H). These results suggested that the *mr1* palea decreased from the early stage of floral organ development.

### Expression of Marker Genes in the Floret

**FIGURE 4:**
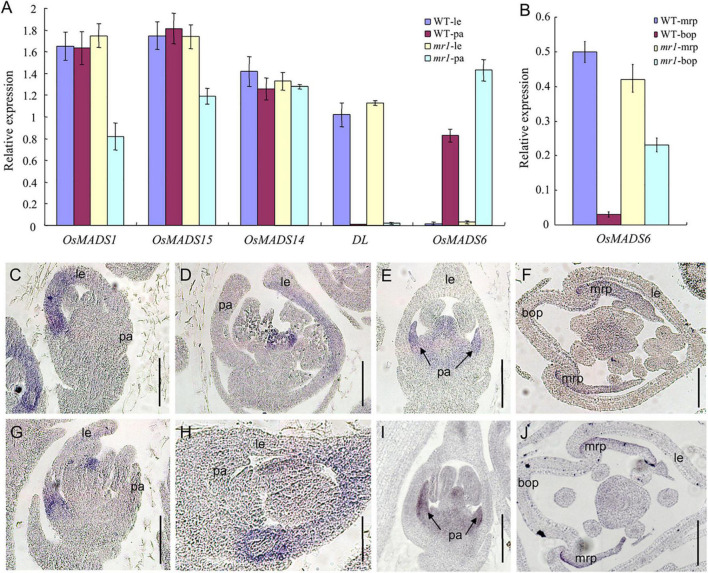
Expression of floral organ genes in the wild-type and the *mr1* mutant. **(A)** Relative expression f genes in the wild-type and the *mr1* floral organs. **(B)** Relative expression f genes in the bop and mrp of wild-type and the *mr1* mutant. **(C,D)** *DL* expression in the wild-type floret. **(E,F)** *OsMADS6* expression in the wild-type floret. **(G,H)** *DL* expression in the *mr1* floret. **(I,J)** *OsMADS6* expression in the *mr1* floret. le, lemma; pa, palea; mrp, marginal region of palea; bop, body of palea. Bars, 50 μm.

To further examine whether the identity of palea has changed, we used qRT-PCR to investigate the expression levels of several marker genes, such as the hull (palea and lemma) marker genes *OsMADS1*, *OsMADS14*, and *OsMADS15*, the lemma marker gene *DROOPING LEAF* (*DL*), and the mrp marker gene *OsMADS6* in the palea and lemma of the wild-type and *mr1* mutant. The results exhibited that the *OsMADS14* expression showed no significant difference between the wild-type and *mr1* mutant (Figure 4A). The expression patterns of *OsMADS1* and *OsMADS15* in the *mr1* lemmas were consistent with that of wild-type lemmas, respectively, while the expression levels of *OsMADS1* and *OsMADS15* in the *mr1* paleae were obviously reduced than that of wild-type paleae, respectively (Figure 4A). *DL* showed no expression difference in the wild-type and *mr1* mutant, while an increase expression of *OsMADS6* was found in the *mr1* mutant (Figure 4A). And, we detailedly detected the expression of *OsMADS6* in the bop and mrp. Compared with the wild-type, the *OsMADS6* transcript was increased in the bop of *mr1* mutant, whereas its expression was not obviously altered in the mrp of *mr1* mutant (Figure 4B). The lower expressions of *OsMADS1* and *OsMADS15* in the *mr1* paleae may be caused by the small bop, while the higher expression of *OsMADS6* in the *mr1* paleae was due to the enlarged mrp.

Next, we further investigate the temporal and spatial expression patterns of the *DL* and *OsMADS6* gene in the wild-type and *mr1* mutant florets by *in situ* hybridization.

In the wild-type, *DL* expression was found in the lemma and pistil at stage SP7, then *DL* mRNAs were found in the lemma at stage Sp8 (Figures 4C,D,G,H). In contrast, we observed no obvious differences of *DL* expression between the wild-type and *mr1* mutant at similar stages (Figures 4C,D,G,H). At stages Sp7 and Sp8, in flowers of the wild-type, *OsMADS6* showed similar expression patterns with the *mr1* mutant; its signals were present in the mrp and pistil (Figures 4E,F,I,J). In general, these results indicated that the identity of mrp was not disturbed in the *mr1* mutant, and the lemma had the similar identity in the wild-type and *mr1* mutant.

### Genetic Analysis

The *mr1* mutant was crossed with Nipponbare, all F_1_ plants appeared the wild-type phenotype, showing that this mutation was a recessive trait. The phenotype of *mr1* was separated in F_2_ population. The total number of F_2_ population was 2,367, including 1,809 normal plants and 558 mutant plants (Supplementary Table 1). The separation ratio was 3.242:1. By the chi-square test, the separation ratio was consistent with the theoretical ratio of 3:1 (Supplementary Table 1). Thus, we reached a conclusion that the trait of *mr1* mutant was controlled by a single recessive gene.

### Mapping of *MR1*

**FIGURE 5:**
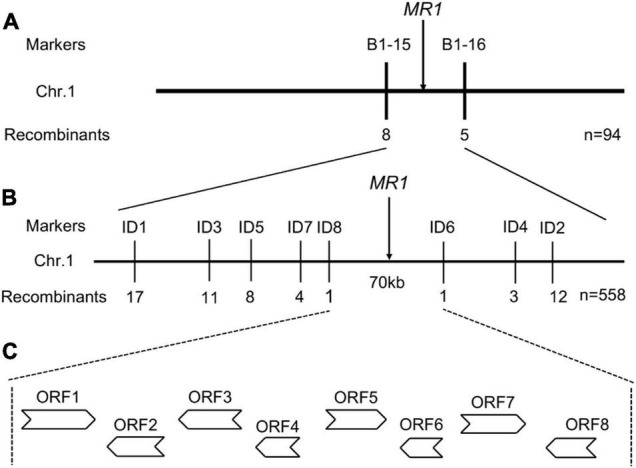
Fine mapping of the *MR1* gene. **(A)** Genetic linkage map for the primary location of the *MR1* gene using 94 F_2_ recessive plants. **(B)** Fine physical map of the *MR1* gene using 558 F_2_ recessive plants. **(C)** ORFs in the target region. ORFs indicate open reading frames.

In order to identify the *MR1* gene, we performed a map-based cloning method. A total of 558 mutant plants in F_2_ population of *mr1*/Nipponbare cross were used for gene mapping. A total of 240 pairs of SSR markers with an average distribution of 12 chromosomes were selected for polymorphism analysis, 112 of which showed polymorphisms between two parents, SKZ and Nipponbare. Using these 112 pairs of SSR markers for linkage analysis, we found that the marker B1-15 on chromosome 1 was obviously linked to the *mr1* phenotype. Then 558 mutant plants in the F_2_ population were investigated with this marker, and 32 recombinants were found. Other 20 SSR markers, which were located near the B1-15, were examined with the same approach. Then, the *MR1* gene was initially located between the two polymorphic markers, B1-15 and B1-16, and they had 32 and 25 recombinants, respectively (Figure 5A). To further determine the location of the *MR1* gene, 16 new in/del primers were developed based on the sequences downloaded from the rice genomic databases (Nipponbare for *japonica* and 93-11 for *indica*), and eight of them exhibited the polymorphisms. All 558 mutants were screened with these eight pairs of primers, and the recombinants were 17, 11, 8, 4, 1, 1, 3, and 12, respectively (Figure 5B). Finally, the *MR1* gene was located in about 70 kb region between the markers ID8 and ID6 (Figure 5B). In this region, eight open frames were shown, and no known genes were reported (Figure 5C and Supplementary Table 3). ORF1 encoded a protein phosphatase 2C. ORF2 encoded a ZOS1-11-C2H2 zinc finger protein. ORF3, ORF4, and ORF8 encoded expression proteins. ORF5 and ORF7 encoded translation initiation factors. ORF6 encoded an OsFBX16-F-box domain protein. We next sequenced the nucleotide sequences of all genes. Unfortunately, we examined the DNA sequence, and failed to identify the nucleotide changes between wild-type and *mr1* mutant.

### The Expression Regulation of Genes Involved in Palea Development

**FIGURE 6:**
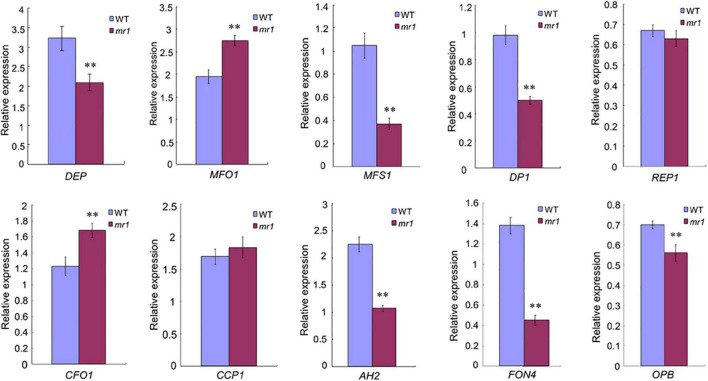
Expression analysis of palea identity-associated genes by qRT-PCR. **Significant difference at *p* < 0.01 compared with the wild-type by Student’s *t*-test. Error bars indicate SD.

In consideration of the *mr1* mutant displayed defects in palea development, we detected whether *MR1* regulates the expression levels of known genes related to palea development in rice. We examined MADS-box genes comprising class A gene (*DEP*/*OsMADS15*) and class E gene (*MFO1/OsMADS6*), and other palea-related genes (*MFS1*, *CFO1*, *CCP1*, *DP1*, *REP1*, *OPB*, *AH2*, and *FON4*). We used qRT-PCR to analyze the expression levels of these genes in the wild-type and the *mr1* mutant young panicles less than 2 cm (Figure 6). Most tested genes displayed significant changes in the *mr1* mutant except *REP1* and *CCP1* (Figure 6). These results indicated that the reduced palea in the *mr1* mutant may be associated with the changes of expression levels of these palea development-related genes, and *MR1* determined the palea fate by possibly promoting *MFO1* and *CFO1*, or suppressing *DEP*, *MFS1*, *DP1*, *OPB*, *AH2*, and *FON4*.

### *mr1* Changed Grain Size and Quality

**FIGURE 7:**
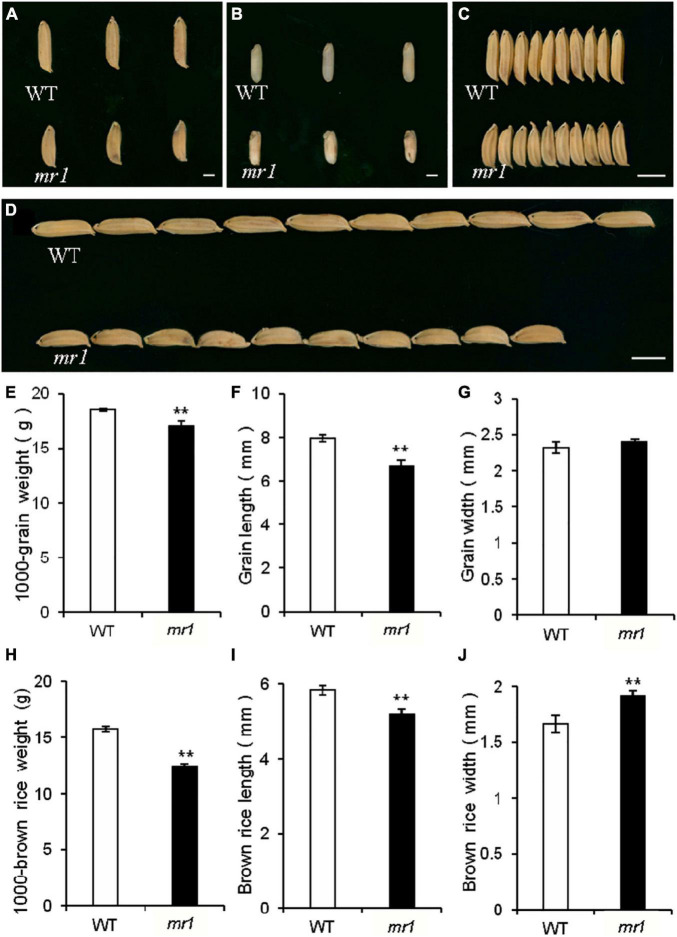
Grains of the wild-type and the *mr1* mutant. **(A)** Grains of the wild-type and *mr1* mutant. **(B)** Brown rice of the wild-type and *mr1* mutant. **(C–D)** Grains of the wild-type and *mr1* mutant. **(E–J)** Length, width, and weight of grains and brown rice in the wild-type and *mr1* mutant. Scale bar, 2 mm in **(A,B)**; 5 mm in **(C,D)**. **Significant difference at *p* < 0.01 compared with the wild-type by Student’s *t*-test. Error bars indicate SD.

The length and 1,000-grain weight of grains in the *mr1* mutant were significantly decreased, but the grain width was not changed than that of the wild-type, respectively (Figures 7A–G). However, the length, width, and 1,000-grain weight of brown rice in the *mr1* mutant were significantly reduced than that of wild-type, respectively (Figures 7E–J). We examined several genes that determined grain size by modulating cell proliferation and expansion (Yu et al., 2020; Hao et al., 2021). Compared with the wild-type, the transcript levels of *BIG1*, *BIG2*, *GW2*, *GS3, GL3, GIF1*, and *BSG1*, were significantly altered in the *dls1* mutant (Supplementary Figure 1), implying that the mutated *MR1* disturbed the expressions of grain size-related genes.

**FIGURE 8:**
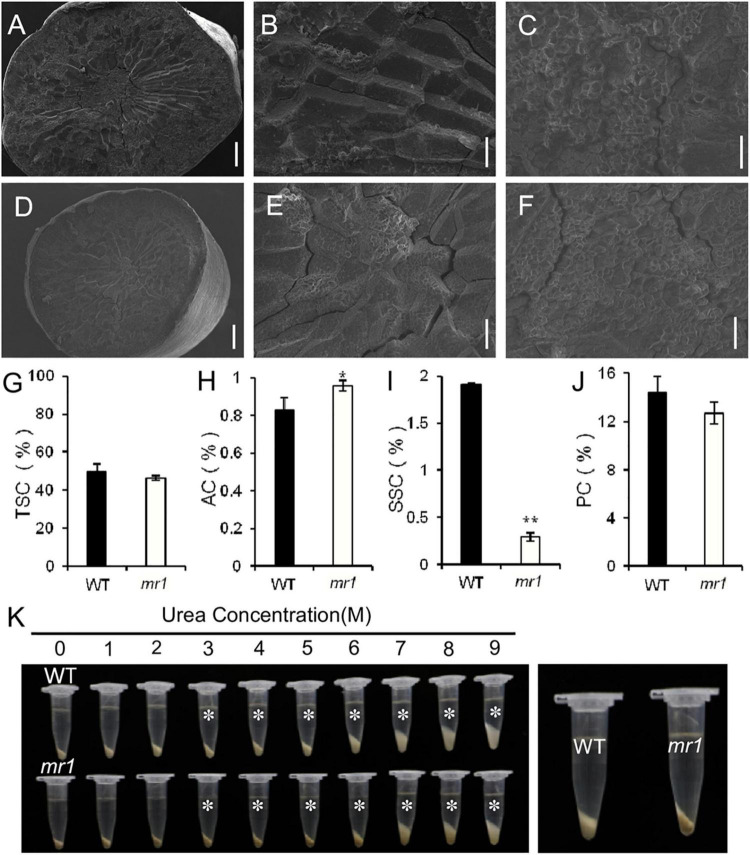
Characteristics of grain and starch in the wild-type and *mr1* mutant. **(A–F)** SEM analysis of transverse sections of brown rice in the wild-type and *mr1* mutant. **(A–C)** Brown rice of the wild-type. **(D–F)** Brown rice of the *mr1* mutant. **(G–J)** Total starch, amylose, soluble sugar, and protein content in the wild-type and *mr1* mutant. **(K)** Gelatinization characteristics of starch in urea solutions (1–9 M). Asterisks indicate the starch of *mr1* endosperm is more difficult to gelatinize in 3–9 M urea solution than that of WT. The most significant difference was observed for 3 M urea (right). Bars, 500 μm in **(A,D)**; 100 μm in **(B,E)**; 20 μm in **(C,F)**. *Significant difference at *p* < 0.05 compared with the wild-type by Student’s *t*-test. **Significant difference at *p* < 0.01 compared with the wild-type by Student’s *t*-test. Error bars indicate SD.

We also investigated several physicochemical properties of starch (Figures 8A–K). In the *mr1* mutant, the amylose content was increased, and the soluble sugar content was decreased that of the wild-type, respectively (Figures 8H,I). And, the total starch and total protein contents of *mr1* grains were not influenced than that of the wild-type, respectively (Figures 8G,J). The starch solubility in urea solutions was also measured to examine the gelatinization properties. The powered rice samples were mixed with various concentrations (0–9 mol L^–1^) of urea solutions. The results exhibited that there are obvious differences in (3–9) mol L^–1^ urea between the wild-type and *mr1* starch, and the *mr1* starch was more difficult to gelatinize than the wild-type starch (Figure 8K). We could conclude that the physicochemical properties of starch were obviously different between the wild-type and *mr1* endosperm, indicating that *MR1* could affect grain quality.

## Discussion

### *MR1* Regulates Palea Development

As an indispensable part of developmental biology, flower development has been widely studied, and the ABCDE model, which successfully interprets the formation of four whorls in flower, has also been widely accepted. Nevertheless, grasses have highly specialized flowers and inflorescence structures, which makes it hard to explain the process of flower development with the knowledge of other model plants. In this study, the palea of *mr1* mutant was reduced while all the other floral organs (pistil, lodicule, and lemma) were still normal except stamen number. Polymorphic markers were used to locate the gene related to the mutation on the long arm of chromosome 1, and there is no report about the related genes of mutation character is located near this position. Therefore, we suggested that *MR1* may be a new gene related to the development of palea in rice.

The origin and identity of the palea and lemma remain a topic of heated discussion. There are some genes that affect the development of both palea and lemma. For example, *DEGENERATED HULL1* (*DH1*) encodes a protein with a LOB domain. In the *dh1* mutant, about most florets could not develop normal lemma and palea, but degenerated into membranous or fibrous organs, and with the loss of lodicules, stamens, and pistils (Li et al., 2008). *TRIANGULAR HULL1* (*TH1*) gene encodes a DUF640 domain transcription factor, the development of palea and lemma was affected both in transverse and longitudinal direction, and they became smaller and thicker in the *th1-1* mutant (Li et al., 2012). *Oryza sativa JAGGED* (*OsJAG*) encodes a C2H2 zinc finger domain transcription factor, the loss of function leads to the malformation of all floral organs, such as lemma and palea degenerated and bent inward, lodicules elongated, pistils deformed, and all stamens became pistil-like organs (Duan et al., 2010). In our study, the *mr1* mutant specially affected palea development, but the lemma development is not influenced, so we speculate that although these two organs have some similarities in morphology, they have different identities and may be regulated by different genetic pathways. Firstly, the palea was usually considered to be a prophyll, and the flower occurs in the axil of it (Ren et al., 2013), and the lemma was widely considered to be a true bract surrounding the spikelet (Luo et al., 2005; Zheng et al., 2015). Secondly, they were different in structure and size. The palea had three vascular bundles, while the lemma had five, so the palea was smaller than the lemma. And the palea was composed of bop and mrp, while the lemma had only one component. In addition, there were several genes, such as *OsMADS15*, *MFO1*, *DP1*, and *CFO1*, showing that there are lots of genes which only affected the development of palea, but not that of lemma. All these facts are consistent with our idea.

Furthermore, in a barley mutant called *leafy lemma*, its lemma was completely transformed into a vegetative leaf with ligule, blade, and sheath, while the palea and other floral organs did not change at all, suggesting that the lemma in grass may not be part of the floret perianth (Pozzi et al., 2000). And in the same barley mutant, the palea and lemma exhibited different phenotypes, indicating that palea and lemma are not homologous in barley. In rice, there is a floral organ identity gene *SUPERWOMAN1* (*SPW1*), it belongs to class B genes and is homologous to *APETALA3* in *Arabidopsis*. The lodicules of *spw1* mutant transformed into palea-like organs instead of lemma (Nagasawa et al., 2003), which indicating that the palea of rice may be equivalent to the sepal of eudicot in evolution. Here, in the *mr1* florets, the palea was reduced, while the lemma maintained its identity, which seemed to confirm the view that the palea and lemma have different origins. However, some other researchers believed that the palea, and even the lemma, should be equivalent to the sepals of eudicot (Ambrose et al., 2000; Goto et al., 2001). Now, there is not corresponding mutants to support such a hypothesis, so it is still not confirmed.

There are various bop or mrp defective mutants in previous studies. *MOSAIC FLORAL ORGANS1* (*MFO1*) and *CHIMERIC FLORAL ORGANS1* (*CFO1*) both belong to the MADS-box genes, their mutants showed enlarged and silicified mrp, which makes the mutant palea look like bop or lemma-like organ (Ohmori et al., 2009; Sang et al., 2012). *RETARDED PALEA1* (*REP1*) belongs to *TCP* gene family, in the *rep1* mutant, the bop was decreased and the development of it was delayed, but in the *REP1* overexpression plants, the mrp overgrew (Yuan et al., 2009). *DEPRESSED PALEA1* (*DP1*) encodes an AT-hook DNA-binding protein, *MULTI-FLORET SPIKELET1* (*MFS1*) encodes an AP2/ERF protein, both the bop of *dp1* and *mfs1* mutant was completely lost, and only two marginal regions were preserved (Jin et al., 2011; Ren et al., 2013). In recent studies, *ABNORMAL HULL2* (*AH2*) and *FLORAL ORGAN NUMBER4* (*FON4*) were proved to be able to identify the bop (Ren et al., 2019a,b). In the *ah2* and *fon4* mutants, the bop size was changed, while the mrp was not altered. In our study, detailed observations revealed that the *mr1* mutant bore an enlarged mrp and a reduced bop, which suggests that the bop was partly converted to mrp, and *MR1*was required for bop and mrp identity. Molecular evidences also showed that the small bop of *mr1* mutant indeed had the mrp identity. These findings suggested that the bop and mrp were homologous organs and a fused organ. Together, more mutants will be needed to further confirming the relationship of the lemma, bop, and mrp.

In this study, the *MR1* gene was fine mapped on chromosome 1 with a physical distance of 70 kb. In this region, there were eight genes, and none of them had been cloned to be related to palea development. But according to the results of sequencing and alignment of these ORF genes between the SKZ and *mr1* mutant, we failed to identify the *MR1* gene. So we speculated that the *mr1* mutant phenotypes might be caused by the intergenic enhancer or repressor and the alteration of promoter sequence or gene expression in the target region. Obviously, this hypothesis needed more experimental evidences to be verified. For instance, we can sequence and compare the promoter regions or 5′ and 3′ UTR sequences, and detect the expression levels of candidate genes. We believe that the further cloning and functional analysis of the *MR1* gene would be helpful to enrich the molecular mechanism of rice palea development, and provide abundant evidences to reveal the relationship between its hull (palea and lemma) and the floral organs of eudicots, as well as the evolutionary relationship between the bop, mrp, and lemma.

### *MR1* Affects Grain Size and Quality

We also observed the *mr1* mutant produced smaller defective grains compared with the wild-type. Several known genes that control grain size by modulating cell proliferation and expansion have been reported. *BIG1*, *BIG2*, *GW2*, *GS3, GL3, GIF1*, and *BSG1* transcripts were changed in the *mr1* mutant than in the wild-type. In addition, we found that the *mr1* grains had low SSC, and high AC and the *mr1* starch was more difficult to gelatinize than that of the wild-type. These findings revealed that the mutation of *MR1* reduced the grain size and quality, which may be caused by defective paleae. Taken together, these results suggested that *MR1* had a possible effort to improve the grain yield and quality.

## Data Availability Statement

The original contributions presented in the study are included in the article/Supplementary Material, further inquiries can be directed to the corresponding author.

## Author Contributions

DR designed the research. DR, WX, WL, XY, and DZ performed the research. DR and WX wrote the manuscript. All authors contributed to the article and approved the submitted version.

## Conflict of Interest

The authors declare that the research was conducted in the absence of any commercial or financial relationships that could be construed as a potential conflict of interest.

## Publisher’s Note

All claims expressed in this article are solely those of the authors and do not necessarily represent those of their affiliated organizations, or those of the publisher, the editors and the reviewers. Any product that may be evaluated in this article, or claim that may be made by its manufacturer, is not guaranteed or endorsed by the publisher.

## Footnotes

1
http://www.gramene.org
