# Supplementary material for: Fine Mapping of Rice Specific MR1, a Gene Determines Palea Identity
Source: Front Plant Sci. 2022 May 24;13:864099. doi: 10.3389/fpls.2022.864099 (PMC9171376; doi:10.3389/fpls.2022.864099)
Supplement: Supplementary file 1 [file Data_Sheet_1.PDF]

SUPPORTING INFORMATION

Supplemental Figure 1

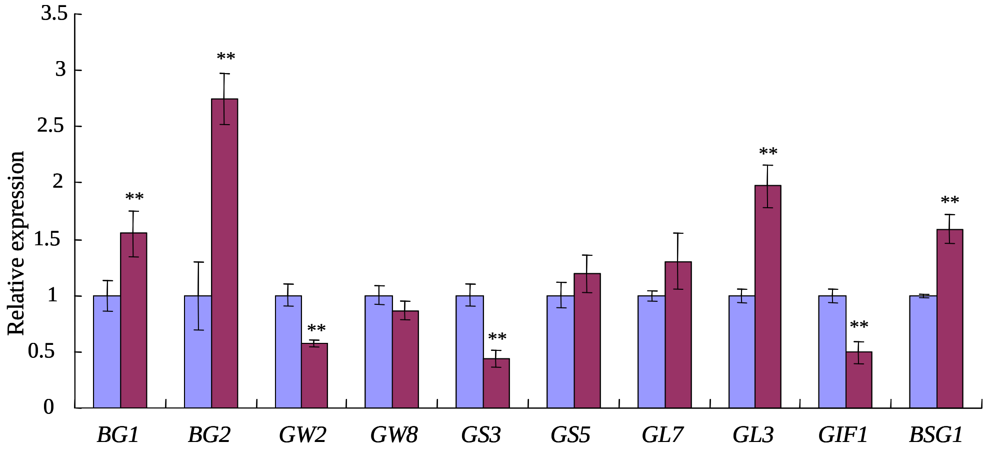

Supplemental Figure 1. Relative expressions of cell expansion and proliferation related genes in the wild type and *mr1* mutant. **Significant difference at $P < 0.01$ compared with the wild type by Student's *t*-test. Error bars indicate SD.

Supplemental Tables

Supplemental Table 1. Genetic analysis of *mr1* traits.

Cross combination	F2		χ2
	wt	*mr1*	
mr1/Nipponbare	1809	558	2.567
Nipponbare/*mr1*	762	221	3.324

Supplemental Table 2. Primers used in the study.

Purpose	Primer name	Sequence
Mapping	B1-15F	GACTGCATGACATCCTGGTG
	B1-15R	GACAGCTTCATCACGCACC
	B1-16F	TGCGGGACCTATGTGGGAC
	B1-16R	ATCTGGAGCTGGATGGGTTCG
	ID-6F	CCCTCTAAAGCCGGTGAACT
	ID-6R	GGCAAACCTAAATTCCGGTAG
	ID-8F	GCTAATGGATTGCTCCGTTT
	ID-8R	TGTTCAGAGTTTGGGTTCAGT
qRT-PCR	*OsMADS1*-1F	CCAAGCCACTCTTCTTGTTCG
	OsMADS1-1R	TGATGGTGAGCATGAGGGTG
	OsMADS14-1F	CCATTAACGAGCTTCAACGG

	OsMADS14-1R	TGGTATGGATCTGAAGCCTCC
	OsMADS15-1F	AGTACGCCACTGACTCCAGG
	OsMADS15-1R	TGCTGGCCCCTCACATTC
	OsMADS6-1F	ATGGGGAGGGGAAGAGTT
	OsMADS6-1R	CGCTGAGTGGTCCAAGAT
	DL-1F	CCCATCTGCTTACAACCGCTT
	DL-1R	GTTGGAGGTGGAAACCGTCG
	ACTIN-1F	AGCAACTGGGATGATATGGA
	ACTIN-1R	CAGGGCGATGTAGGAAAGC
	BG1-1F	GATGGAGAGCGACGAGGAC
	BG1-1R	GCAATGGCGGCGAAGTTC
	BG2-1F	GGCGTCTTGGGCGACTTCGT
	BG2-1R	CCACCGTGTCCGTCCCTCTA
	BSG1-1F	TTCTCCGTGGCTTGAAGTAT
	BSG1-1R	AATCGGTTTCTGAGGTGGTA
	GIF1-1F	CATGTACCAGCCGACGTTTG
	GIF1-1R	GCTCTCAACAACCGACCTGTC
	GL3-1F	GCTCAAGGTCACCTGATCACTC
	GL3-1R	GAACGACCACAAGATCTCTGC
	GL7-1F	CCCCTAGCATCGACACCAAG
	GL7-1R	CGGGTTCCAGCACTCCTCT
	GS3-1F	CGGAAGAACTCCTGATCCATTC
	GS3-1R	CACTTGCTCTGCACAAACAGC
	GS5-1F	GTTCTCGGTACTGCGTGGAAG
	GS5-1R	ACTCCACAAACCTCCCAGCA
	GW2-1F	CAGCCACCCAGTATGGACTTC
	GW2-1R	ACATGCTTCCACCAGCAATGT
	GW8-1F	GGGATGATCAAAACCGAGGAG
	GW8-1R	GTCAGAGGTGGAGCCAACGA
In Situ Hybridization	*OsMADS6*-1F	GACGCAACTGATGATGGAACAAG
	*OsMADS6*SP6-1R	AGATTTAGGTGACACTATAGAAGACGCA GAAGGTGCAAACAGC
	*DL*H-1F	GGGCCGCAATGGATCTCGTG
	*DL*SP6-1R	AGATTTAGGTGACACTATAGAATCACAA CGAAGGGTGCTCT

Supplemental Table 3. Predicted ORFs and annotations in the target region on chromosome 1.

ORF	Gene name	Annotation
ORF1	*LOC_Os01g40094*	Protein phosphatase 2C
ORF2	*LOC_Os01g40110*	ZOS1-11-C2H2 zinc finger protein
ORF3	*LOC_Os01g40120*	Expressed protein

ORF4	*LOC_Os01g40140*	Expressed protein
ORF5	*LOC_Os01g40150*	Eukaryotic translation initiation factor 5B
ORF6	*LOC_Os01g40160*	OsFBX16-F-box domain containing protein
ORF7	*LOC_Os01g40170*	Translation initiation factor
ORF8	*LOC_Os01g40180*	Expressed protein
